# Insight on how fishing bats discern prey and adjust their mechanic and sensorial features during the attack sequence

**DOI:** 10.1038/srep12392

**Published:** 2015-07-21

**Authors:** Ostaizka Aizpurua, Antton Alberdi, Joxerra Aihartza, Inazio Garin

**Affiliations:** 1 Department of Zoology and Animal Cell Biology. Faculty of Science and Technology. University of The Basque Country, UPV/EHU. Sarriena z.g., E-48940 Leioa, The Basque Country

## Abstract

Several insectivorous bats have included fish in their diet, yet little is known about the processes underlying this trophic shift. We performed three field experiments with wild fishing bats to address how they manage to discern fish from insects and adapt their hunting technique to capture fish. We show that bats react only to targets protruding above the water and discern fish from insects based on prey disappearance patterns. Stationary fish trigger short and shallow dips and a terminal echolocation pattern with an important component of the narrowband and low frequency calls. When the fish disappears during the attack process, bats regulate their attack increasing the number of broadband and high frequency calls in the last phase of the echolocation as well as by lengthening and deepening their dips. These adjustments may allow bats to obtain more valuable sensorial information and to perform dips adjusted to the level of uncertainty on the location of the submerged prey. The observed ultrafast regulation may be essential for enabling fishing to become cost-effective in bats, and demonstrates the ability of bats to rapidly modify and synchronise their sensorial and motor features as a response to last minute stimulus variations.

Fish are an occasional to common component of the diet of several echolocating bat species worldwide^1^. Fishing most likely emerged as a modification of the specific hunting technique named *trawling*, which consists of capturing insects lying on the water surface using the hind feet^2^. Compared to trawling, fishing may present a greater challenge for bats, because echolocation is of limited value for detecting prey under water, due to the strong attenuation of sound at the air-water interface^3^. However, fishing bats seem to rely mostly on echolocation to detect and locate their prey^4^,^5^.

The capture technique used by bats for fishing differs from that used for hunting insects^5^, which means that bats are not only able to detect different prey, but also to discern between insects and fish. However, the underlying process of prey recognition is still unknown. Insects and fish differ in their morphological features; while insects are winged and articulated, fish are soft-bodied. Both prey types also differ in their behaviour. Insects remain stationary in the water surface offering a continuous detectable acoustic cue, whilst the pelagic surface-feeder fish consumed by bats^6^,^7^,^8^,^9^,^10^ produce ripples and splashes, and expose parts of their body only momentarily producing transient cues^5^. Hence, bats may recognise their prey based on morphology, since echoes produced by fish are presumably different from echoes produced by insects, or may rely on the transience of the produced cues.

The transitory nature of the acoustic cues produced by fish entails further difficulties for bats when detecting and identifying the prey. Regardless of the mechanism used to discern prey types, if bats detect fish stimulus from long distances they have to perform the capture attempt blindly, as the fish would most likely disappear long before the bat reaches its location. Additionally, if bats just relied on temporality of the cue to discern prey, the moment the fish submerges—thus revealing its nature—would be crucial for triggering one or the other hunting technique because of limits to the reaction ability of bats^11^.

When fishing, bats modify their flight and dip features by increasing the flight-speed and performing longer and deeper dips^5^. Regarding echolocation, when bats start approaching a target they increase the rate of echolocation calls to avoid pulse-echo overlap, rapidly updating the sensorial information^12^. This ultimate stage of the echolocation sequence is called the *terminal phase*, and its function is not still fully understood. In trawling species, including fishing bats, the *terminal phase* is divided in two parts: *buzz I* and *buzz II*^12^. *Buzz I* is comprised of echolocation calls with broader bandwidth and higher frequency than *buzz II* calls, which provide greater sensorial capabilities with respect to information intake and higher beam directionality^13^,^14^. The *terminal phase* is flexible, as its entire length varies depending on the foraging habitat, target size and difficulty of the task performed^5^,^15^,^16^. In a previous work, we described the general differences between insect hunting and fishing, and concluded that the internal components of the *terminal phase* differ depending on the prey type, using longer *buzz I* and shorter *buzz II* when fishing compared to when catching insects^5^. However, the functional differences between *buzz I* and *buzz II* still remain controversial^14^ and the main questions integral for understanding how insectivorous bats have been able to accomplish this trophic shift were not addressed: how do bats manage to discern between fish and insects? Which are the sensorial basis and advantages that lead bats to shift their echolocation pattern?

Aiming to resolve these questions, we designed three new field experiments using free-flying fishing long-fingered bats, *Myotis capaccinii*. The first experiment was designed to identify the type of surface stimuli fishing bats recognise by analysing the frequency of positive responses to different stimuli. The objective of the second experiment was to determine whether bats use prey morphology or the temporality of the produced cue to decide how to attack the target by measuring changes in the dip and echolocation pattern between stationary and submerging fish. Finally, to gain insight on how bats react to last minute prey disappearance and identify the specific sensorial and motor reasons why bats modify their *terminal phase* composition we analysed variations in the hunting technique in response to different fish disappearance-times.

The results of this study not only shed light on how bats are able to detect and identify fish, but also advance our knowledge of the general sensorial capabilities of echolocating bats when detecting and targeting different prey types.

## Results

### Target recognition experiment

The frequency of responses of *M. capaccinii* bats to the experimental treatments differed across stimuli. Almost all the hunting attempts (99%) were directed at stationary and temporary targets (69% and 30% respectively). Bats rarely responded to ripple stimuli (2 out of 261 attempts), and not a single attack occurred in the control patch.

### Target identification experiment

**Figure 1:**
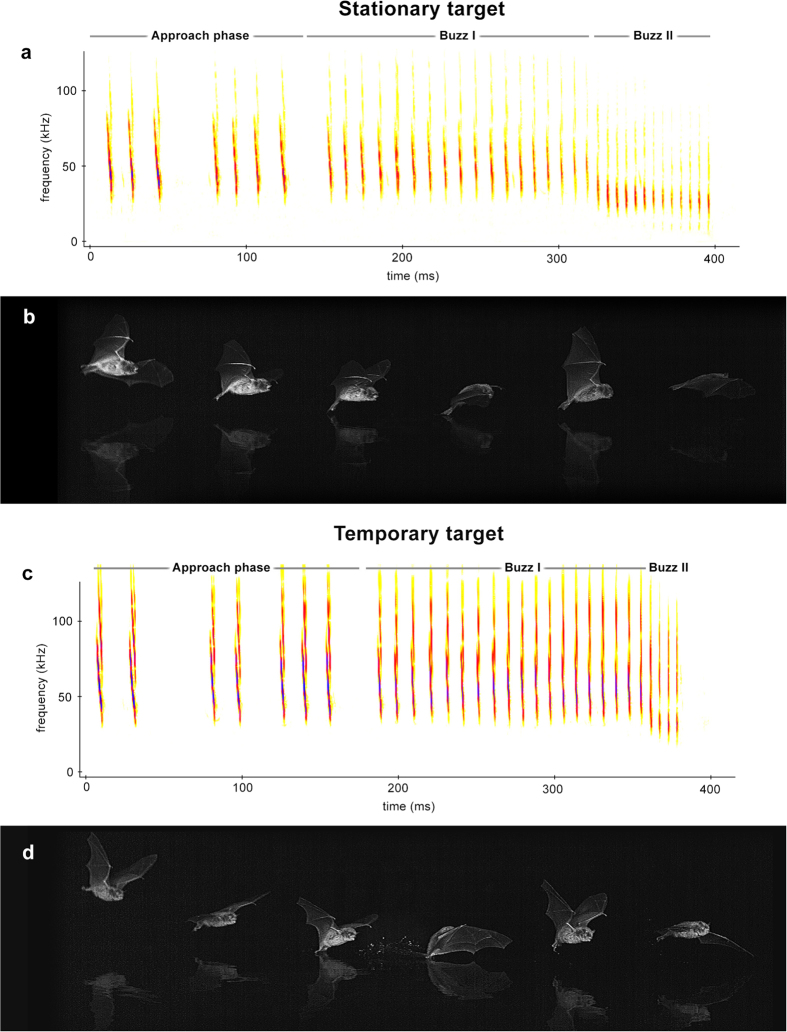
Graphical representation of the technique the bats use when attacking stationary (**a**–**b**) and temporary targets (**c**–**d**). The spectrograms (**a**) and (**c**) show the terminal echolocation phase characteristics. The frame-sequences (**b**) and (**d**) show the dip features. Note that the spectrograms and frame-sequences shown are not synchronised. Synchronised images, sound and spectrograms are provided in Supplementary Movie 1.

All recordings of capture attempts made on stationary and temporary targets included a *terminal phase* (*n* = 74 and *n* = 69, respectively), even though 8.6% of the temporary target attempts lacked *buzz II*. The duration and number of pulses in the *terminal phase* as a whole were not significantly different between the capture attempts made on the two target types (duration: *t*-test, t = −0.1, *P* = 0.913; number of pulses: *t*-test, t = 1.6, *P* = 0.106) (Table 1). However, *buzz I* contained more pulses and was thus longer when attempting to capture the temporary target than for the stationary target (number of pulses: *t*-test, t = −3.2, *P* = 0.001; duration: *t*-test, t = 2.3, *P* = 0.023). Conversely, *buzz II* contained fewer pulses and was shorter for the temporary target than for the stationary target (number of pulses: Mann-Whitney, U = 878.5, *P* < 0.001; duration: *t*-test, t = −5.5, *P* < 0.001) (Table 1 and Fig. 1a–c). Therefore when bats responded to the stationary target, *buzz I* and *buzz II* calls were more balanced than during attempts made on the temporary target (see Supplementary Movie 1).

In both treatments, the bats dragged their feet through the water to catch the target, but when attacking the temporary target the duration of the dips was almost double that observed during attacks on stationary target (*t*-test, t = 6.6, *P* < 0.001; Table 1 and Fig. 1b–d). The feet insertion depth was also different (χ^2^(1) = 36.6, *P* < 0.001). When aiming at the stationary target, dragging was performed mainly with the bats’ toes—59.4% of cases—and the whole hind foot was never submerged. In contrast, when attacking the temporary target more than half of the hind foot was submerged in 86.8% of the cases, sometimes even up to the ankle (see Supplementary Movies 1 and 2 for details of capture technique and feet insertion).

### Target disappearance experiment

**Figure 2:**
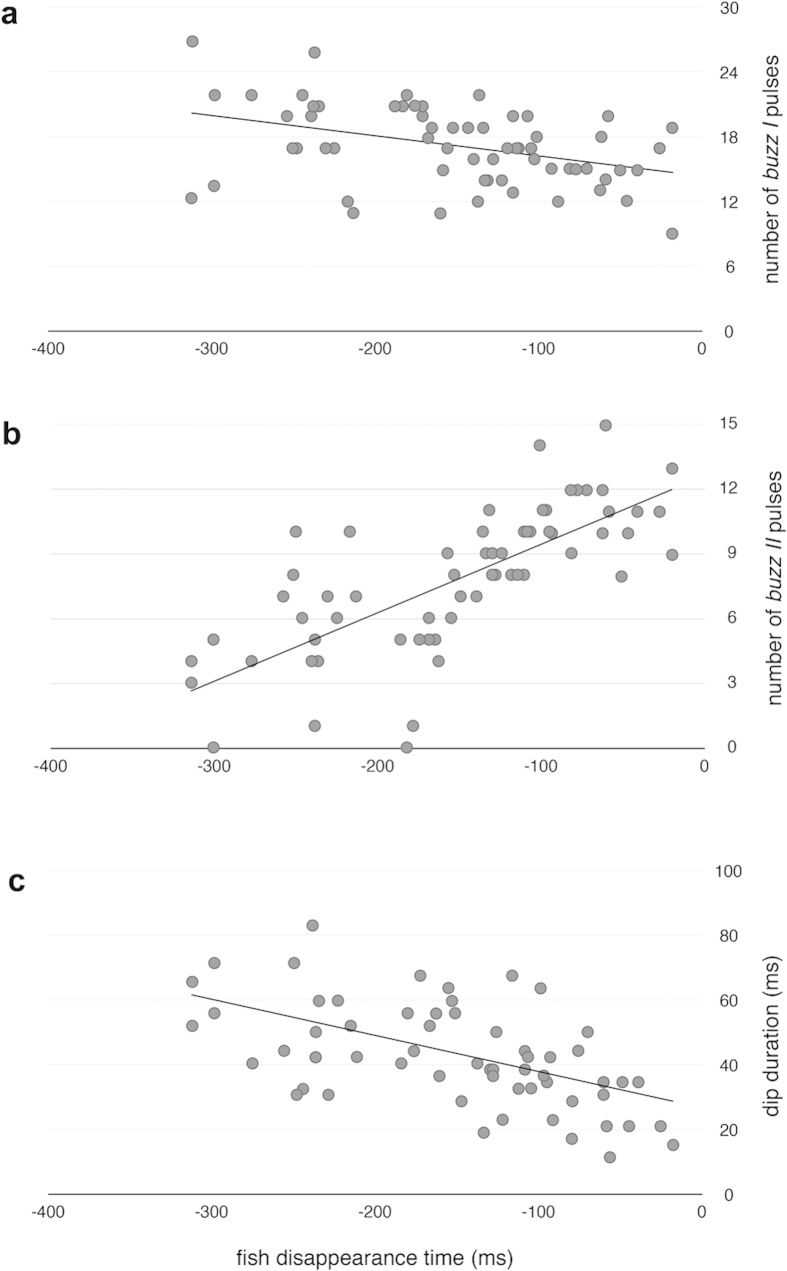
Relationship between fish disappearance time and (**a**) number of *buzz I* pulses, (**b**) number of *buzz II* pulses and (**c**) dip duration. Feet insertion is used as a reference for target disappearance time.

When the bats attacked the temporary target, the number of pulses of both buzzes and the dip duration were all significantly related to the timing of prey submersion (number of pulses in *buzz I*: Pearson’s r = −0.40, *P* = 0.002; number of pulses in *buzz II*: Pearson’s r = 0.73, *P* < 0.001; dip duration: Pearson’s r = −0.57, *P* < 0.001). Specifically, the earlier the prey disappeared, the higher the number of *buzz I* pulses, the lower the number of *buzz II* pulses and the longer the dip (Fig. 2).

## Discussion

In the present work we were able to resolve several issues that remained unanswered in previous studies, gaining insight on how wild long-fingered bats manage to recognise, identify and capture fish, and how and why bats rapidly adjust and synchronize their motor and sensorial features in response to last minute stimuli variations.

The stimulus recognition experiment showed that when bats are fishing they almost respond exclusively to targets protruding above the water. This is in accordance with Siemers *et al.*^17^ who suggested that isolated echo-reflecting objects on an acoustic *mirror,* like water, might be considered as prey by bats. Bats detected and attacked targets rising even slightly above the surface using echolocation. They precisely located the target and even if the prey disappeared during the very early phase of the capture process (>250 ms before prey contact) the bats continued their attack toward the last location of the item. The task could be accomplished with the aid of the ripples produced by the fish when submerging, which the bats could use as footprints indicating the spot where the previously detected fish had disappeared. Ripples may offer an information cue for bats due to the higher variance in echo amplitude that they produce compared to smooth water surfaces, though they are only noticeable at close ranges^18^. As the amplitude and overall structure of the received echo is fainter at narrower angles, the ability to perceive ripples also diminishes when the bat is further away from the target. Accordingly, the resulting response to the experimental treatment with the ripple stimuli was extremely low.

The results obtained in the target identification experiment demonstrate that target disappearance, instead of its morphology, is the stimulus triggering a change in the hunting technique. Stationary targets were attacked using short and superficial dips and an even composition of buzz types, while target disappearance elicited longer and deeper dips, as well as a larger proportion of *buzz I* calls relative to the length of the *terminal phase*. In fact, in almost 10% of the attempts on temporary targets *buzz II* was missing. It is important to note that observed differences between stationary and temporary target captures are almost identical to the differences Aizpurua *et al.*^5^ observed between insect hunting and fishing attempts of *M. capaccinii*. Since in this study we used morphologically identical fish targets in both treatments, the features that distinguish the response of bats were only related to the disappearance pattern of the prey. In fact, the attacks on stationary fish were identical to insect hunting attacks^5^. Thus, bats most probably discern fish from insects based on the temporality of the acoustic cue than their morphological features. Additionally, the target disappearance experiment revealed that there was a significant correlation between the time of target disappearance and the modification of the *terminal phase* and dip features: the earlier the disappearance of the prey along the final stages of the capture process, the higher the number of pulses in *buzz I,* the lower the number of *buzz II* pulses and the longer the dip. All these features reveal a shift from insect hunting to fishing-like attack pattern. When the prey disappears late in the attack sequence, the attack resembles an insect hunting, while as the prey disappears sooner, the sensorial and motor features shift to what Aizpurua *et al.*^5^ defined as an fishing-like attack. Seemingly, bats are able to process the information received during the attack process, to modify the temporal components of the buzzes and change the capture technique during the attack.

The lengths of *buzz I* and *buzz II* are regulated depending on the time of disappearance of the target. *Buzz II* plays an active role with continuously detectable prey, but is more trivial when attempts are made on temporary prey that are undetectable from a certain moment onwards—like live fish. Jakobsen & Surlykke^14^ proposed that the role of the lower frequency in *buzz II* is to achieve a wider detection angle during the final part of prey pursuit in order to counteract the insects’ evasive manoeuvres. Accordingly, long-fingered bats hunting continuous targets would have kept long *buzz II* to effectively face putative terminal displacements of the prey. Conversely, *buzz II* was reduced and sometimes even omitted when the prey disappeared in the initial stages of hunting. These findings suggest that *buzz II* loses its importance because there is no detectable prey and therefore evasive movements are not expected. However, bats did not interrupt their signal emission but instead extended *buzz I*. Bats need to keep track of the location from where the fish disappeared over an entirely smooth surface without apparent references, sometimes for as long as for 300 ms. In this context, the ripples generated by the submerging fish could serve as an indicator of its location. Bats could extend *buzz I* to continue emitting high frequency pulses with enhanced beam directionality and echo strength^19^, which, in turn, would allow them to better perceive the variance in echo amplitude of the ripples^18^ produced by the submerged fish in the last instances of the capture process. The ability to regulate the internal components of the *terminal phase* seems to play a key role in fishing, but whether this capacity is a feature specific to fishing bats, to all trawling bats or to echolocating bats in general will need to be addressed in the future.

Regarding dip characteristics, we observed that when the prey disappears under the water bats perform longer and deeper dips, i.e. they sample a larger volume of water plausibly using tactile stimuli. While the modifications in echolocation could be an innate response of bats to target disappearance and thus not a specific response to fish prey, the modification of the dip pattern necessarily entails an interpretation of the situation. Lengthening and deepening the drag produces a high energetic cost, so the motor reaction we observed makes sense only if bats interpret that the previously detectable prey is somewhere under the water close to the surface, and is worth spending more energy because they can be rewarded by a highly energetic prey. Further, bats performed longer dips as the target disappeared earlier, likely adjusting their dip to the degree of uncertainty regarding the prey’s presumed location. The ability to regulate the dipping behaviour may play an important role in making fishing more efficient, as the regulation of dip length in relation to the degree of prey location uncertainty allows the bats to save a considerable amount of time and energy.

This paper shows that the long-fingered bats’ default hunting technique is the insect capture mode, characterised by short and shallow dips and an even *buzz I* to *buzz II* ratio. However, bats interpret target disappearance during the attack attempt as a submerged target, so they lengthen and deepen their dip and skew the buzz-ratio toward *buzz I*. The sooner the prey disappears under the water, the divergence from the default mode increases, demonstrating that bats are able to process the echo information received during the attack and regulate the temporal components of their buzzes and their physical attack based on that information. The reduction in *buzz II* or its complete omission when capturing temporary targets provides evidence that *buzz II* is emitted in order to respond to last minute prey movements, as it is largely reduced when the missing prey cannot be tracked anymore. The high flexibility of the echolocation *terminal phase*, as well as the fast and coupled sensorial and motor response revealed in our experiments, most likely played a key role in the evolutionary success of echolocating bats, and in particular in the trophic niche expansion of fishing species.

## Methods

### Study area

The field study was carried out in June-July 2012 in “La Sella” golf course (Dénia, Valencian Community, Eastern Iberian Peninsula). To date this is the only location where fishing behaviour by the long-fingered bat, *Myotis capaccinii*, has been recorded and studied in the wild^5^,^10^.

### Experimental setup

We used a normal-speed digital video camera (Sony HDR550, Sony Corporation, Tokyo, Japan) and infrared light torches (IREL-45, ECV Video Seguridad S.A., Sabadell, Catalonia) to record free-flying wild bats in high activity spots (>15 bats simultaneously foraging; Supplementary Movie 3). Species identification in the field was based on visual observations and echolocation call analysis (D1000X and BatSound software—Pettersson Elektronik AB, Uppsala, Sweden) following Aizpurua *et al.*^5^.

For the stimuli recognition experiment, we produced three stimuli, mimicking natural conditions: (1) small surface ripples, (2) a stationary fish breaking the water surface with its upper lip, and (3) a vertically moving fish that broke the surface intermittently and created small ripples around the breaking point. As a control treatment, we recorded bat activity over an unmodified pond patch of 25 × 25 cm. A submerged rod placed perpendicular and at a known distance to the microphone was used to set the different stimuli conditions. The rod was split in four equal parts, one for each stimulus and the control. The position of the stimuli along the rod was randomly changed each day. We used a submerged propeller to produce small surface ripples without any target breaking the water surface. To represent a stationary target we used a dead eastern mosquitofish *Gambusia holbrooki* (the species *M. capaccinii* consumes in the wild^10^) that was tethered from the abdomen with small tweezers. As a temporary target we also used a dead eastern mosquitofish, but tethered with a fishing line that would cause the target to either protrude from the water’s surface for one second or submerse it for two seconds. Fish were caught with a hand-net in the same pond where the bats usually fish and sacrificed by cervical dislocation^20^. They were placed under water with the upper lip breaking the surface. This experiment was performed using a normal-speed video camera (Sony HDR550, Sony Corporation, Tokyo, Japan) and infrared torches (IREL-45, ECV Video Seguridad S.A., Sabadell, Catalonia) to survey the frequency of bat attacks on each stimulus (Supplementary Movie 3).

In the target identification experiment we analysed changes in echolocation and attack pattern between stationary and temporary targets. We combined a low-light high-speed video camera (HiSpec, Fastec imaging Corporation, USA) capable of recording near-IR light with an ultrasound detector (D1000X, Pettersson Elektronik AB, Uppsala, Sweden). High-speed videos were recorded at 500 frames per second onto a Window platform laptop using Fastec software (Fastec Imaging Corporation, USA), and were assisted by infrared lighting (IREL-45, ECV Video Seguridad S.A., Sabadell, Catalonia). The camera was placed approximately 20 cm above the water surface, and all the studied attacks were perpendicular to the position of the camera. Audio recordings were made at a high sampling rate (350 kHz) in real-time mode at 16-bit, and were stored as WAV files. The sound and video inputs were synchronised using an electronic clapper (for more information see Aizpurua *et al.*^5^). This allowed for fine-scale temporal correlation of visual and acoustic information to define the *terminal phase* start and end time in the video. We used the moment of contact between the bats’ feet and the water as a temporal reference point to align the capture sequences. For all time-based analyses, we defined this instant as time zero, and we present all the time information relative to this point of reference. Therefore, events occurring before water contact scored negative time values.

In the target disappearance experiment the temporary target was randomly submersed at different times (19.4–313.9 ms before the prey contact point) along the bat target pursuit flight to investigate how *M. capaccinii* responds to the disappearance of a target and to assess the ability of bats to switch the capture mode. We analysed variation in echolocation and attack patterns using the same methodological framework as in the target identification experiment.

### Analysis of the recordings

We analysed 261 responses of *M. capaccinii* to the stimuli recognition experiment, and 136 and 69 synchronised recordings for the target identification and target disappearance experiments, respectively.

Sound analyses were performed with the software BatSound (Pettersson Elektronik AB, Uppsala, Sweden). We analysed only the *terminal phase* of the echolocation call sequence. The *terminal phase* was defined as a continuous sequence of calls emitted by the bat just before a capture attempt and directly after a pre-buzz pause^21^,^22^,^23^. This *terminal phase* is divided into two parts: *buzz I* and *buzz II*. Sound duration and pulse interval are continuously reduced throughout *buzz I*, but the peak call frequency is kept at a steady frequency, while *buzz II* is characterised by a distinct drop in frequency in the entire call structure^12^. For each recording we measured the total duration and pulse number of the *terminal phase*, *buzz I* and *II* duration and number of pulses, and the percentage of each buzz out of the total echolocation pulses produced (% of pulses).

Video recordings were analysed using Fastec software (Fastec Imaging Corporation, USA). For each attack, we directly measured from the high-speed video recordings the total dip duration and feet insertion depth (the degree to which the hind feet are introduced into the water), which was classified into four categories: (1) no contact with water, (2) touching the water with the toes, (3) introduction of half of the foot into the water and (4) submersion of more than half of the foot into the water.

### Statistical analysis

All statistical analyses were performed using the software SPSS 20.0.0 (SPSS Inc., Chicago, IL, USA). Parameters confirmed as normally distributed by a Kolmogorov-Smirnov test were analysed using one-way Student *t*-tests (α = 0.05). For parameters not fulfilling the assumption of normality, we used Mann-Whitney *U*-test alongside Pearson’s Chi-Squared test (χ^2^) to compare frequencies. The relationships between fish disappearance moment and different variables (number of *buzz I* pulses, number of *buzz II* pulses and dip duration) were analysed using the Pearson product-moment correlation coefficient.

### Ethics statement

Fish capture and handling protocols met the guidelines for treatment of animals in research and teaching^24^, met Spanish legal requirements, and validated by the Ethics Committee for Animal Welfare of the University of the Basque Country (Refs CEBA/220/2012/AIHARTZA and CEBA/221/2012/AIHARTZA).

## Additional Information

**How to cite this article**: Aizpurua, O. *et al.* Insight on how fishing bats discern prey and adjust their mechanic and sensorial features during the attack sequence. *Sci. Rep.* **5**, 12392; doi: 10.1038/srep12392 (2015).

## Supplementary Material

Supplementary Information

Supplementary Information

Supplementary Information

Supplementary Information

## Figures and Tables

**Table 1:** General features of the *terminal phase* and dip duration measured during *Myotis capaccinii*attacks on the two experimental targets.

**Terminal phase**	**Stationary target**	**Temporary target**
**Total number of pulses**	26.24 ± 4.01	25.18 ± 3.53
**Total duration (ms)**	192.12 ± 33.63	193.19 ± 34.80
***Buzz I***: duration (ms)^*^	131.51 ± 31.63	149.94 ± 35.98
***Buzz I***: number of pulses^*^	15.00 ± 3.55	17.19 ± 3.84
***Buzz I:%*** of pulses^*^	56.30 ± 7.59	73.30 ± 14.09
***Buzz II***: duration (ms)^*^	55.72 ± 10.91	38.24 ± 13.71
***Buzz II***: number of pulses^*^	11.24 ± 1.90	7.44 ± 3.71
***Buzz II:%*** of pulses^*^	43.70 ± 7.59	26.70 ± 14.09
***Terminal phase*** **start (ms)**	−220.38 ± 44.59	−207.63 ± 42.53
***Terminal phase*** end (ms)*	−25.55 ± 15.54	−11.80 ± 11.94
Dip duration (ms)*	25.52 ± 10.33	42.59 ± 15.65

Feet insertion is used as a reference for *terminal phase* start and end calculations. Asterisks indicate significant differences between the two targets (*P* < 0.05). Results are presented as means ± standard deviation.
