# Supplementary material for: Insight on how fishing bats discern prey and adjust their mechanic and sensorial features during the attack sequence
Source: Sci Rep. 2015 Jul 21;5:12392. doi: 10.1038/srep12392 (PMC4508917; doi:10.1038/srep12392)
Supplement: Supplementary Information [file srep12392-s4.pdf]

Supplementary Information

Insight on how fishing bats discern prey and adjust their mechanic and sensorial features during the attack sequence

Ostaizka Aizpurua[1*], Antton Alberdi[1], Joxerra Aihartza[1] & Inazio Garin[1]

[1]Department of Zoology and Animal Cell Biology. Faculty of Science and Technology. University of The Basque Country, UPV/EHU. Sarriena z.g., E-48940 Leioa, The Basque Country

[*]E-mail: ostaizka.aizpurua@ehu.eus

Supplementary Movie 1 | Details of the dip and echolocation features when attacking stationary and temporary targets. The video shows synchronised video, echolocation spectrogram and audio at a 1/20 reproduction-speed.

Supplementary Movie 2 | Details of the feet insertion when attacking stationary and temporary targets.

Supplementary Movie 3 | The experimental setup for the target recognition experiment. Note the high number of free-flying bats in the experimental area.
